# Exploring the metabolite composition and biological activities of *Thymus canoviridis* via volatile and non-volatile fraction analysis

**DOI:** 10.3389/fnut.2025.1675586

**Published:** 2026-01-14

**Authors:** Ezgi Ersoy, Selami Ercan, Ercan Çınar, Sevcan İzgi, Gülsüm Tuneğ, Gökhan Zengin, Emel Mataracı Kara, Yeter Yeşil, Hasan Şahin, Mehmet Boğa, Esra Eroğlu Özkan

**Affiliations:** 1 Department of Pharmacognosy, Faculty of Pharmacy, Biruni University, Istanbul, Türkiye; 2 Department of Chemistry, Faculty of Arts and Science, Batman University, Batman, Türkiye; 3 Department of Nursing, School of Health Sciences, Batman University, Batman, Türkiye; 4 Department of Medical Biology and Genetics, Faculty of Medicine, Gaziantep University, Gaziantep, Türkiye; 5 Department of Medicinal Biology, Faculty of Medicine, Dicle University, Diyarbakir, Türkiye; 6 Selçuk University, Faculty of Science, Department of Biology, Konya, Türkiye; 7 Department of Pharmaceutical Microbiology, Faculty of Pharmacy, Istanbul University, Istanbul, Türkiye; 8 Department of Pharmaceutical Botany, Faculty of Pharmacy, Istanbul University, Istanbul, Türkiye; 9 Department of Pharmacognosy, Faculty of Pharmacy, Dicle University, Diyarbakir, Türkiye; 10 Department of Analytical Chemistry, Faculty of Pharmacy, Dicle University, Diyarbakir, Türkiye; 11 Department of Pharmacognosy, Faculty of Pharmacy, Istanbul University, Istanbul, Türkiye

**Keywords:** *Thymus canoviridis*, carvacrol, rosmarinic acid, enzyme inhibition, antimicrobial activity, food preservation

## Abstract

*Thymus canoviridis*, an endemic species in Türkiye, was studied for its chemical composition and biological activities, with emphasis on food-related applications. GC-MS analysis of the essential oil from aerial parts revealed an exceptionally high carvacrol content (99.9%), highlighting its potential as a natural preservative. LC-MS/MS profiling of ethanol extracts (aerial and root) identified rosmarinic acid as the dominant phenolic (885.53 ± 7.25 μg/g in aerial part; 721.08 ± 6.14 μg/g in root), along with notable levels of apigenin and quinic acid. The aerial extract showed higher total phenolic (114.39 ± 1.86 mg PEs/g) and flavonoid contents (47.80 ± 0.94 mg QE/g) than the root extract. *In vitro* antioxidant assays revealed strong activity for both extracts: the root was more active in DPPH (IC_50_: 24.32 ± 0.84 μg/mL) and CUPRAC (A_0.5_: 9.99 ± 0.02 μg/mL), while the aerial part extract was superior in ABTS (IC_50_: 8.36 ± 0.05 μg/mL). The essential oil exhibited outstanding ABTS (IC_50_: 0.43 ± 0.02 μg/mL) and CUPRAC (A_0.5_: 3.36 ± 0.07 μg/mL) activity. Enzyme inhibition assays showed strong α-glucosidase inhibition (IC_50_: 683.35 ± 3.75 μg/mL) by the oil and selective butyrylcholinesterase inhibition (69.61 ± 1.84% at 200 μg/mL). Antimicrobial tests demonstrated significant activity against *Candida tropicalis* (MIC: 19.53 μg/mL) and *Staphylococcus aureus* (MIC: 39.06 μg/mL). Taken together, *T. canoviridis* represents a promising source of bioactive compounds with antioxidant, antimicrobial, and enzyme-inhibitory properties suitable for functional food and clean-label preservation applications.

## 1 Introduction

The growing demand for natural and health-promoting ingredients in the food and nutraceutical industries has fueled the exploration of underutilized aromatic and medicinal plants. Members of the genus *Thymus* (Lamiaceae), comprising over 200 species primarily distributed across the Mediterranean region, have long been recognized for their culinary, aromatic, and therapeutic properties (1, 2). Many species of the genus *Thymus* (Lamiaceae) have long been consumed across Mediterranean cuisines—particularly in Türkiye and Greece—as herbal teas, aromatic spices in meat and dairy dishes, and natural preservatives in local food preparations (3, 4). However, despite extensive research on commonly used *Thymus* taxa such as *T. vulgaris* and *T. serpyllum*, certain endemic species from Anatolia remain chemically and biologically underexplored, representing untapped resources for the development of novel bioactive ingredients.

*T. canoviridis* Jalas is an endemic species distributed in limited areas of Türkiye (5). Although relatively less studied, this species is known to be used by local populations in infusions and as a seasoning herb in meat-based dishes, similar to other culinary *Thymus* taxa (3). Despite its traditional culinary use, comprehensive scientific studies investigating its phytochemical composition and biological functionality remain limited—particularly regarding (i) the comparative chemical composition of its aerial and root parts, (ii) the integration of volatile and non-volatile metabolite profiles, and (iii) the evaluation of its enzyme inhibitory mechanisms relevant to metabolic and neuroprotective health.

One of the distinguishing features of the *Thymus* genus is its chemotypic diversity, often characterized by essential oil profiles dominated by either thymol or carvacrol, two structurally related monoterpenic phenols with distinct bioactivities and sensory profiles (6). In the present study, *T. canoviridis* essential oil was found to contain an exceptionally high proportion of carvacrol, exceeding 99% of its volatile content. This unusually high carvacrol concentration suggests that *T. canoviridis* could serve as a valuable natural source of carvacrol for potential applications in phytotherapy, food preservation, and the development of functional ingredients. To our knowledge, such a high-purity carvacrol has not been previously reported for *T. canoviridis*, making it an important candidate for both phytochemical standardization and functional food innovation.

Carvacrol has gained significant attention in recent years due to its multifaceted biological properties, including antioxidant, antimicrobial, anti-inflammatory, and enzyme-inhibitory effects. These characteristics make it a valuable candidate for applications in functional foods, bioactive packaging, and natural preservatives (7, 8). Moreover, the interest in carvacrol has surged since the COVID-19 pandemic, owing to its supportive role in respiratory health and immune regulation, further promoting its incorporation into dietary supplements and phytotherapeutic formulations (9).

Phenolic compounds and essential oil components from *Thymus* species have been increasingly studied for their enzyme inhibitory activities, particularly against α-glucosidase, urease, acetylcholinesterase, and tyrosinase—enzymes linked to diabetes, neurodegenerative diseases, and skin disorders (10–13). The integration of such natural enzyme inhibitors into functional food systems has emerged as a promising strategy for metabolic health regulation and anti-aging nutrition.

In this first comprehensive study of *T. canoviridis*, we present an integrated metabolite and bioactivity profiling covering both volatile and non-volatile fractions. The essential oil composition was analyzed by GC-MS, while phenolic constituents of ethanol extracts from aerial and root parts were identified and quantified using LC-MS/MS. The antioxidant, antimicrobial, and enzyme inhibitory activities of these extracts were also evaluated. By combining chemical characterization with functional assays, this study expands the current phytochemical understanding of *T. canoviridis* and provides a scientific basis for its valorization as a bioactive and functional ingredient within the food and nutraceutical sectors.

## 2 Materials and methods

### 2.1 Chemicals and instruments

Phytochemical analysis of the aerial and root extracts, along with the essential oil of *T. canoviridis*, was conducted using LC-MS/MS (Shimadzu, Kyoto, Japan) and GC-MS (Agilent Technologies Inc., Santa Clara, CA, USA). Antioxidant, antimicrobial, and enzyme inhibitory activities were measured using a BioTek PowerWave XS microplate reader and a UV–Vis spectrophotometer. All analytical-grade reagents and standards used in the assays were obtained from Merck (Germany), Sigma-Aldrich (Germany), Applichem (Germany), and Fluka (Germany).

### 2.2 Plant material

The aerial parts and roots of *T. canoviridis* were collected during the flowering stage in June 2014 from Kemaliye, Erzincan province (Türkiye), located in Eastern Anatolia. The plant material was harvested by Assoc. Prof. Dr. Yeter Yeşil and Prof. Dr. Mehmet Boga. Taxonomic identification was confirmed by Assoc. Prof. Dr. Yeter Yeşil. A voucher specimen (ISTE: 104454) has been deposited at the Herbarium of Istanbul University, Faculty of Pharmacy. The collection site is characterized by a continental climate, with hot and dry summers and cold winters. The plant material was harvested from calcareous, rocky, and well-drained soil typical of the region's mountainous terrain.

### 2.3 Preparation of essential oil and extracts

#### 2.3.1 Essential oil extraction

Aerial parts of *T. canoviridis* (100 g) were subjected to hydrodistillation with 500 mL of distilled water for 3 h using a Clevenger-type apparatus. The essential oil obtained was dried over anhydrous sodium sulfate and diluted with dichloromethane (1:3, v/v) prior to GC-MS analysis. A 1 μL aliquot was injected into the GC-MS system.

#### 2.3.2 Preparation of ethanolic extracts

The plant was separated into aerial (11.24 g) and root (12.77 g) parts, ground with a high-speed blender, and macerated with ethanol (3 × volume) for 3 × 24 h (72 h). The pooled filtrates were evaporated to dryness under vacuum at 40 °C. Dried extracts were stored at −20 °C. For LC-MS/MS analysis, extracts were dissolved in methanol to a final concentration of 0.25 μg/mL and filtered through a 0.2 μm filter.

### 2.4 GC-MS analysis of essential oil

The essential oil was analyzed using an Agilent 7890A GC system coupled with a 5975 MSD. An HP-Innowax FSC column (60 m × 0.25 mm, 0.25 μm film thickness) was used for separation. The injector and detector were set at 250 and 280 °C, respectively. The oven temperature was programmed from 60 °C (1 min) to 190 °C at 20 °C/min (hold 60 min), then to 220 °C at 1 °C/min (hold 10 min). Helium was used as carrier gas (1 mL/min). Compound identification was based on retention indices calculated using co-injected n-alkanes (C8–C30) and mass spectral matching against NIST 05, Wiley 8, and in-house libraries. Quantification was expressed as relative peak areas. Methodology was adapted from Zengin et al. (14).

### 2.5 LC-MS/MS analysis of phenolic compounds

Phenolics in ethanol extracts were analyzed using a Shimadzu Nexera UHPLC system coupled with tandem MS. Separation was achieved using a C18 Inertsil ODS-4 column (150 × 4.6 mm, 3 μm) at 40 °C. Mobile phases consisted of water (5 mM ammonium formate + 0.1% formic acid) (A) and methanol with same additives (B). The gradient was: 0 min−40% B; 20 min−90% B; 24 min−40% B; 29 min—end. Flow rate: 0.5 mL/min; injection volume: 4 μL. Conditions were adapted from Ertaş et al. (15).

### 2.6 Determination of total phenolic and flavonoid contents

Total phenolic and flavonoid contents were determined using Folin–Ciocalteu and aluminum chloride colorimetric methods, respectively (16, 17). Results were expressed as μg pyrocatechol equivalents (PEs) and μg quercetin equivalents (QEs):

- Abs = 0.0409 × pyrocatechol (μg) + 0.0581 (*R*^2^ = 0.9924).
- Abs = 0.0325 × quercetin (μg) – 0.0601 (*R*^2^ = 0.9984).

### 2.7 Antioxidant activity assays

Antioxidant capacity was evaluated using DPPH, ABTS, and CUPRAC assays. Experimental procedures followed Ersoy et al. (18). Results for radical scavenging activities (DPPH and ABTS) are expressed as IC_50_ values (μg/mL), while reducing power in the CUPRAC assay is expressed as A_0.5_ (μg/mL), defined as the concentration of sample giving an absorbance of 0.5. The extracts were tested at concentrations of 10, 25, 50, and 100 μg/mL. Total phenolic and flavonoid contents were also determined and expressed as μg pyrocatechol equivalents (PEs) and μg quercetin equivalents (QEs) per mg extract, respectively.

### 2.8 Enzyme inhibitory activity assays

Enzyme inhibition activities were determined against acetylcholinesterase (AChE), butyrylcholinesterase (BChE) (18), tyrosinase, urease, and α-glucosidase using previously described methods Ersoy et al. (19). Extracts were tested at four concentrations (25, 50, 100, and 200 μg/mL) to establish dose–response relationships. Results were expressed as % inhibition at a fixed concentration (200 μg/mL) for AChE, BChE, tyrosinase, and urease (20), while α-glucosidase inhibition (21) was presented as IC_50_ values (μg/mL).

### 2.9 Antimicrobial activity assays

MICs of extracts were determined using the broth microdilution method against standard bacterial and yeast strains (22–24). Reference strains included: *Staphylococcus aureus* ATCC 29213, *S. epidermidis* ATCC 12228, *Enterococcus faecalis* ATCC 29212, *Pseudomonas aeruginosa* ATCC 27853, *Escherichia coli* ATCC 25922, *Klebsiella pneumoniae* ATCC 4352, *Proteus mirabilis* ATCC 14153, *Candida albicans* ATCC 10231, *C. parapsilosis* ATCC 22019, and *C. tropicalis* ATCC 750. Standard antibiotics (e.g., cefuroxime-Na, ceftazidime, amikacin, clotrimazole) were used as controls. Extract concentrations ranging from 5000 to 1.25 μg/mL were tested.

### 2.10 Statistical analysis

All data were expressed as mean ± standard deviation (SD) of three independent experiments. Statistical analysis was performed using one-way analysis of variance (ANOVA), followed by Tukey's Honestly Significant Difference (HSD) *post-hoc* test to determine differences among extract groups (TCA, TCR, TCEO). A *p*-value less than 0.05 was considered statistically significant. Analyses and visualizations were conducted using Python (v3.10) and the *statsmodels* and *scipy* libraries.

## 3 Results

### 3.1 Volatile compound profile of *T. canoviridis* essential oil (TCEO)

**Figure 1:**
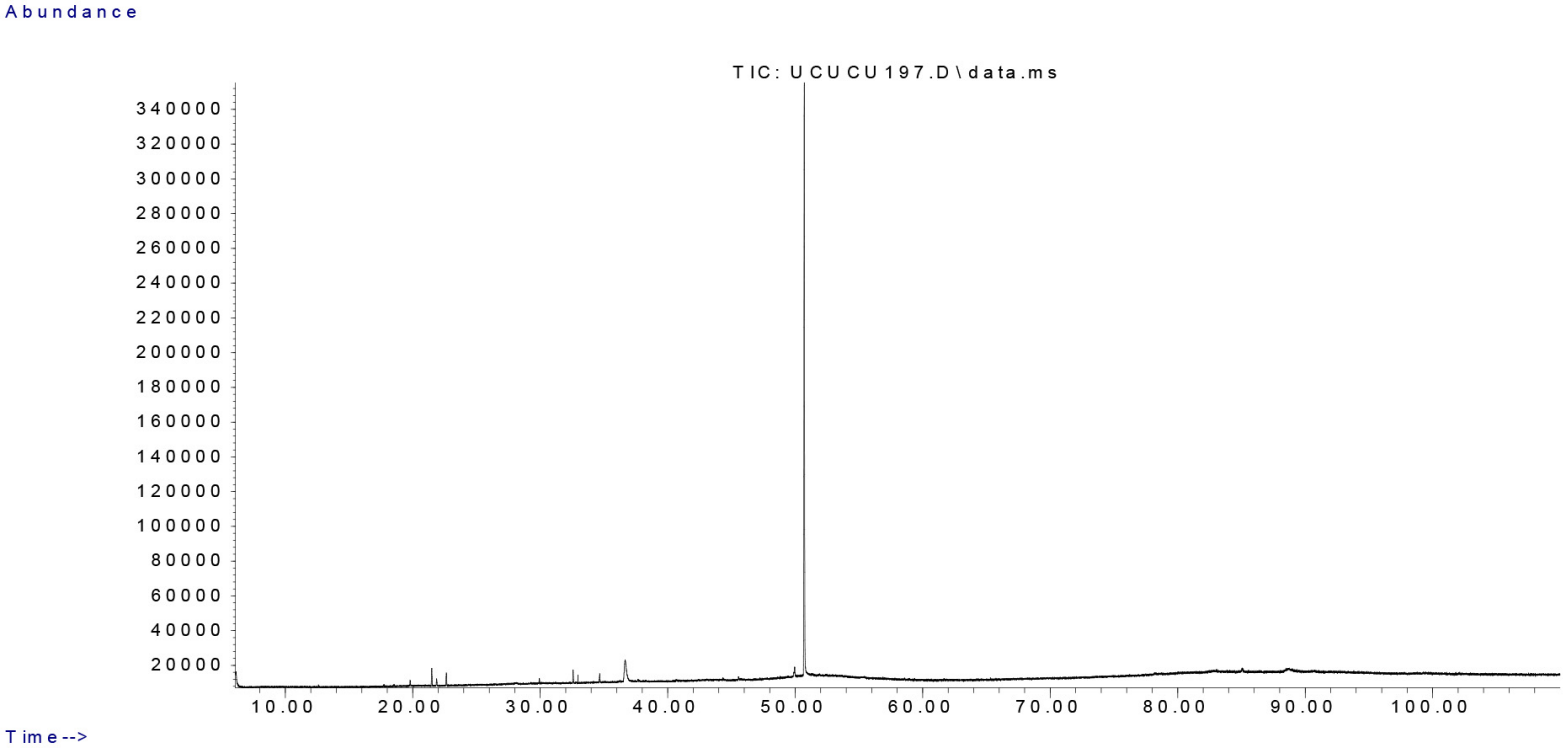
TIC Chromatogram of *Thymus canoviridis* essential oil.

GC-MS analysis of the essential oil from the aerial parts of *T. canoviridis* led to the identification of 99 volatile compounds (Table 1). Among them, carvacrol was identified as the dominant constituent, accounting for 99.9% of the total volatile composition. The total ion chromatogram (TIC) is presented in Figure 1, illustrating the prominent peak corresponding to carvacrol. Other typical *Thymus* volatiles such as thymol, p-cymene, or γ-terpinene were either absent or detected in trace amounts.

**Table 1:** Chemical composition of the essential oil from the aerial parts of *Thymus canoviridis* identified by GC-MS.

**No**	**Compounds**	**RRI^a^**	**(%)**	**No**	**Compounds**	**RRI^a^**	**(%)**
1	Tricyclene	1009	Nd	50	γ-Muurolene	1704	Nd
2	α-Pinene	1023	Nd	51	α-Terpineol	1706	Nd
3	α-Thujene	1026	Nd	52	Ledene	1713	Nd
4	Camphene	1068	Nd	53	Borneol	1715	Nd
5	Undecane	1100	Nd	54	Chamigrene	1723	Nd
6	β-Pinene	1111	Nd	55	Germacrene D	1729	Nd
7	Sabinene	1124	Nd	56	Verbonene	1732	Nd
8	δ-3-Carene	1157	Nd	57	β-Bisabolone	1738	Nd
9	Myrecene	1165	Nd	58	Valencene	1740	Nd
10	α-Phellandrene	1168	Nd	59	α-Muurolene	1740	Nd
11	α-Terpinene	1183	Nd	60	β-Selinene	1743	Nd
12	Limonene	1201	Nd	61	α-Selinene	1747	Nd
13	1,8-Cineole	1211	Nd	62	Geranial	1750	Nd
14	*(Z)*-β-Ocimene	1237	Nd	63	Bicyclogermacrene	1754	Nd
15	γ-Terpinene	1249	Nd	64	Geranyl acetate	1763	Nd
16	*(E)*-β-Ocimene	1255	Nd	65	Citronellol	1768	Nd
17	*p*-Cymene	1276	Nd	66	δ-Cadinene	1773	Nd
18	Terpinolen	1286	Nd	67	γ-Cadinene	1779	Nd
19	6-Methyl-5-hepten-2-one	1343	Nd	68	(E)-α-Bisabolone	1783	Nd
20	3-Octanol	1391	Nd	69	β-Sesquiphellandrene	1783	Nd
21	α-Fenchone	1408	Nd	70	Nerol	1806	Nd
22	α, *p*-Dimethylstyrene	1447	Nd	71	Myrtenol	1806	Nd
23	1-Octen-3-ol	1450	Nd	72	α-Cadinene	1811	Nd
24	Camphenilone	1475	Nd	73	*trans*-Carveol	1846	Nd
25	Longipinene	1479	Nd	74	Calamenene	1854	Nd
26	α-Copaene	1501	Nd	75	*p*-Cymen-8-ol	1861	Nd
27	α-Campholenal	1503	Nd	76	*cis*-Carveol	1877	Nd
28	Chrysanthenone	1523	Nd	77	Geranyl butyrate	1900	Nd
29	β-Bourbonene	1531	Nd	78	Caryophyllene oxide	2017	Nd
30	Camphor	1535	Nd	79	*(E)*-Nerolidol	2051	Nd
31	α-Gurjunene	1542	Nd	80	Ledol	2057	Nd
32	Linalool	1548	Nd	81	Cubenol	2074	Nd
33	β-Cubebene	1549	Nd	82	1-*epi*-Cubenol	2083	Nd
34	1-None-3-ol	1550	Nd	83	Elemol	2095	Nd
35	Linayl acetate	1561	Nd	84	Globulol	2099	Nd
36	Isocamphopinone	1565	Nd	85	Viridiflorol	2108	Nd
37	Fenchol	1592	Nd	86	Spathulenol	2147	Nd
38	Bornyl acetate	1593	Nd	87	*(Z)*-3-hexen-1-yl benzoate	2152	Nd
39	β-Elemene	1601	Nd	88	γ-Eudesmol	2187	Nd
40	Terpinen-4-ol	1612	Nd	89	T-Cadinol	2193	Nd
41	β-Caryophyllene	1614	Nd	90	Thymol	2196	Nd
42	Aromadendrene	1624	Nd	91	T-Muurolol	2208	Nd
43	*trans*-dihydrocarvone	1626	Nd	92	δ-Cadinol	2218	Nd
44	Myrtenal	1651	Nd	93	Carvacrol	2227	99.9^*^
45	Alloaromodendrene	1664	Nd	94	α-Bisabolol	2232	Nd
46	*(Z)*-β-Farnesene	1670	Nd	95	α-Eudesmol	2246	Nd
47	δ-Terpineol	1681	Nd	96	α-Cadinol	2254	Nd
48	α-Humulene	1689	Nd	97	β-Eudesmol	2256	Nd
49	Neral	1698	Nd	98	Selin-11-en-4α-ol	2263	Nd
				99	(*2E,6E*)-Farnesol	2365	Nd
							**99.9**

^a^Relative retention indices calculated against n-alkanes.

Nd, Not detected.

### 3.2 Phenolic composition of ethanol extracts from *T. canoviridis* (TCA and TCR)

The LC-MS/MS analysis of the ethanol extracts from the aerial (TCA) and root (TCR) parts of *T. canoviridis* led to the detection of 21 and 20 phenolic compounds, respectively (Table 2). In total, 25 distinct phenolic compounds were identified across both plant parts, with notable quantitative and qualitative differences between them.

**Table 2:** Quantitative content (μg/g dry extract) of phenolic compounds in ethanol extracts from aerial and root parts of *T. canoviridis* as determined by LC-MS/MS.

**Number**	**Analytes**	**RT^a^**	**Parent ion (m/z)**	**Daughter Ions**	**Ion. Mode**	**Quantification (**μ**g analyte/g extract)**^**b**^	
						**TCA**	**TCR**
**1**	**Coumarin**	17.40	147.05	91.0–103.2	Pos	–	–
**2**	**Hesperidin**	12.67	610.90	303.1–465.1	Pos	40.05 ± 1.05	99.85 ± 2.62
**3**	**p-coumaric acid**	11.53	162.95	119.25–93.25	Neg	41.99 ± 2.17	17.8 ± 0.91
**4**	**o-coumaric acid**	15.45	162.95	119.35–93.25	Neg	–	–
**5**	**Gallic acid**	3.00	168.85	125.2–79.2	Neg	–	–
**6**	**Caffeic acid**	8.80	178.95	135.2–134.3	Neg	722.29 ± 25.57	581.05 ± 20.57
**7**	**Vanillic acid**	8.57	166.90	152.25–108.25	Neg	–	–
**8**	**Salicylic acid**	11.16	136.95	93.3–65.3	Neg	49.52 ± 1.63	18.92 ± 0.62
**9**	**Quinic acid**	1.13	190.95	85.3–93.3	Neg	6404.85 ± 52.52	2858.35 ± 23.43
**10**	**4-OH-Benzoic acid**	7.39	136.95	93.3–65.3	Neg	–	–
**11**	**tr-Ferulic acid**	12.62	192.95	178.3	Neg	89.11 ± 4.40	211.71 ± 10.45
**12**	**Chlorogenic acid**	7.13	353.15	191.2	Neg	165.84 ± 1.14	–
**13**	**Rosmarinic acid**	14.54	359.00	161.2–197.2	Neg	2371.36 ± 169.08	6448.00 ± 459.74
**14**	**Protocatechuic acid**	4.93	152.95	108.3	Neg	271.65 ± 11.16	218.35 ± 8.97
**15**	**Cinnamic acid**	25.61	147.00	103.15–77.3	Neg	–	–
**16**	**Sinapinic acid**	12.66	222.95	208.3–149.2	Neg	–	–
**17**	**Fumaric acid**	1.48	115.00	71.4	Neg	645.65 ± 8.01	382.58 ± 4.74
**18**	**Vanillin**	10.87	151.00	136.3–92.2	Neg	49.19 ± 1.38	70.93 ± 1.98
**19**	**Pyrocatechol**	6.48	109.00	108.35–91.25	Neg	–	–
**20**	**Malic acid**	1.23	133.00	115.2–71.3	Neg	644.00 ± 7.28	477.18 ± 5.39
**21**	**Syringic acid**	9.02	196.95	182.2–167.3	Neg	–	–
**22**	**Hesperetin**	31.76	300.95	164.2–136.2	Neg	11.66 ± 0.66	3.80 ± 0.21
**23**	**Naringenin**	30.68	270.95	151.2–119.3	Neg	1028.79 ± 53.60	40.95 ± 2.13
**24**	**Rutin**	12.61	609.05	300.1–271.1	Neg	17.07 ± 0.27	27.36 ± 0.43
**25**	**Quercetin**	28.17	300.90	151.2–179.2	Neg	–	–
**26**	**Quercitrin**	16.41	447.15	301.15–255.15	Neg	–	–
**27**	**Apigenin**	31.43	268.95	117.3–151.2	Neg	234.16 ± 15.22	15.58 ± 1.01
**28**	**Chrysin**	36.65	252.95	143.3–119.4	Neg	2.08 ± 0.04	5.63 ± 0.11
**29**	**Liquiritigenin**	25.62	254.95	119.25–135.15	Neg	–	–
**30**	**Isoquercitrin**	13.42	463.00	300.15–271.15	Neg	16.24 ± 0.22	1.09 ± 0.01
**31**	**Cosmosiin**	16.59	431.00	268.2–239.2	Neg	760.90 ± 45.43	18.17 ± 1.08
**32**	**Rhoifolin**	16.11	577.05	269.2–211.15	Neg	1451.31 ± 136.57	38.11 ± 3.58
**33**	**Nicotiflorin**	14.68	593.05	285.1–255.2	Neg	8975.52 ± 247.72	645.36 ± 17.81
**34**	**Fisetin**	19.30	284.95	135.2–121.25	Neg	–	–
**35**	**Luteolin**	28.27	284.75	133.2–151.2	Neg	–	–
**36**	**Myricetin**	18.72	317.00	179.15–151.25	Neg	–	–
**37**	**Kaempferol**	31.88	284.75	255.1–117.3	Neg	–	–

^a^RT, Retention time, ^b^Values in μg/g (w/w) of plant extracts.

TCA, *Thymus canoviridis* aerial part; TCR, *Thymus canoviridis* root.

Among the compounds detected, nicotiflorin (8975.52 μg/g), quinic acid (6404.85 μg/g), and rosmarinic acid (2371.36 μg/g) were the most abundant in the aerial part. The TCA extract also contained high levels of naringenin (1028.79 μg/g), cosmosiin (760.90 μg/g), and apigenin (234.16 μg/g), along with caffeic acid (722.29 μg/g) and fumaric acid (645.65 μg/g).

In comparison, the TCR extract was characterized by rosmarinic acid (6,448.00 μg/g), hesperidin (99.85 μg/g), and caffeic acid (581.05 μg/g) as dominant components. Notably, several phenolic acids such as tr-ferulic acid (211.71 μg/g) and protocatechuic acid (218.35 μg/g) were also present in appreciable amounts. Flavonoids, including naringenin, cosmosiin, and nicotiflorin, were significantly less concentrated in the roots compared to the aerial parts.

These findings reflect a distinct partitioning of phenolic compounds between the aerial and root tissues of *T. canoviridis*, underscoring tissue-specific metabolic pathways and functional specialization.

### 3.3 Total phenolic, flavonoid content and antioxidant activity of *T. canoviridis*

The ethanol extracts of the aerial (TCA) and root (TCR) parts of *T. canoviridis*, along with the essential oil (TCEO), were evaluated for their total phenolic and flavonoid contents, as well as antioxidant activity using DPPH, ABTS, and CUPRAC assays (Table 3).

**Table 3:** Total phenolic and flavonoid contents, and antioxidant activity (DPPH, ABTS, and CUPRAC assays) of ethanol extracts from aerial and root parts of *T*. canoviridis^*^.

**Samples**	**Phenolic content (μg PEs/mg extract)^a^**	**Flavonoid content (μg QEs/mg extract)^b^**	**DPPH free radical IC_50_ (μg/mL)**	**ABTS cation radical IC_50_ (μg/mL)**	**CUPRAC A_0.5_ (μg/mL)**
**TCA**	59.56 ± 0.87	35.35 ± 1.05	33.08 ± 0.54	8.36 ± 0.05	10.59 ± 0.04
**TCR**	144.12 ± 2.60	17.35 ± 0.52	24.32 ± 0.84	9.72 ± 0.08	9.99 ± 0.02
**TCEO**	–	–	273.42 ± 1.65	0.43 ± 0.02	3.36 ± 0.07
**BHA** ^**c**^	–	–	7.88 ± 0.20	2.74 ± 0.03	0.63 ± 0.02
**α-TOC** ^**c**^	–	–	16.30 ± 0.79	10.20 ± 0.05	13.38 ± 0.07
**BHT** ^**c**^	–	–	58.86 ± 0.50	3.16 ± 0.06	2.02 ± 0.01

TCA, *Thymus canoviridis* aerial part; TCR, *Thymus canoviridis* root; TCEO, *Thymus canoviridis* essential oil

^*^Values expressed are means ± standard deviation of three parallel measurements (p < 0.05).

^a^PEs, pyrocatechol equivalents (y = 0.0409x + 0.0581, R^2^ = 0.9924).

^b^QEs, quercetin equivalents (y = 0.0325x + 0.0601, R^2^ = 0.9984).

^c^Standard compounds.

The TCR extract exhibited the highest total phenolic content with 144.12 ± 2.60 μg PEs/mg extract, followed by TCEO (64.19 ± 0.83 μg PEs/mg) and TCA (59.56 ± 0.87 μg PEs/mg). In contrast, the TCA extract showed the highest flavonoid content (35.35 ± 1.05 μg QEs/mg), which was roughly double that of the root extract (17.35 ± 0.52 μg QEs/mg). The essential oil had only minor flavonoid presence (4.31 ± 0.38 μg QEs/mg).

Antioxidant assays revealed notable differences in radical scavenging and reducing capacities between the samples. In the DPPH assay, the root extract (TCR) exhibited stronger activity (IC_50_ = 24.32 ± 0.84 μg/mL) compared to the aerial extract (TCA, IC_50_ = 33.08 ± 0.54 μg/mL). However, the essential oil (TCEO) demonstrated the weakest DPPH activity (IC_50_ = 273.42 ± 1.65 μg/mL).

Interestingly, in the ABTS assay, TCEO was the most potent (IC_50_ = 0.43 ± 0.02 μg/mL), outperforming both extracts (TCA: 8.36 ± 0.05 μg/mL, TCR: 9.72 ± 0.08 μg/mL), and even showing comparable results with standard compounds such as BHT and BHA. This strong ABTS activity may be attributed to the hydrophobic antioxidant potential of carvacrol-rich oil.

In the CUPRAC assay, TCR again showed superior reducing ability (A_0.5_ = 9.99 ± 0.02 μg/mL), followed by TCA (10.59 ± 0.04 μg/mL) and TCEO (3.36 ± 0.07 μg/mL). Among standard antioxidants, BHA showed the strongest activity in all assays.

According to the one-way ANOVA followed by Tukey HSD test, the phenolic content of TCR was significantly higher than TCA and TCEO (*p* < 0.001), while the flavonoid content of TCA was significantly higher than TCR and TCEO (*p* < 0.001). TCEO exhibited significantly lower IC_50_ in the ABTS assay compared to TCA and TCR (*p* < 0.001), despite having the lowest total phenolic and flavonoid content. These findings suggest that the ABTS scavenging effect of TCEO may be attributed to non-phenolic compounds present in the essential oil.

These findings highlight a tissue-specific antioxidant profile, with the root extract (TCR) being particularly rich in phenolic acids contributing to DPPH and CUPRAC activity, while the essential oil's hydrophobic nature and carvacrol content make it more effective in the ABTS system.

### 3.4 Enzyme Inhibition activity of *T. canoviridis*

The ethanol extracts from the aerial (TCA) and root (TCR) parts, along with the essential oil (TCEO) of *T. canoviridis*, were evaluated for their inhibitory effects on several clinically relevant enzymes, including acetylcholinesterase (AChE), butyrylcholinesterase (BChE), tyrosinase, urease, and α-glucosidase (Table 4).

**Table 4:** Enzyme inhibition activity of ethanol extracts from aerial and root parts of *T*. *canoviridis*^a^.

**Samples**	**AchE (% inhibition)**	**BchE (% inhibition)**	**Tyrosinase (% inhibition)**	**Urease (% inhibition)**	**α-Glucosidase^c^ (IC_50_, μg/mL)**
TCA	NA	33.93 ± 0.67	NA	NA	954.55 ± 1.48
TCR	NA	13.89 ± 0.62	NA	NA	>1800
TCEO	69.75 ± 1.14	69.61 ± 1.84	–	–	683.35 ± 3.75
Galanthamine^b^	78.92 ± 1.04	78.22 ± 0.58	–	–	–
Kojic acid^b^	–	–	95.05 ± 0.37	–	–
Tiyourea^b^	–	–	–	98.37 ± 0.40	–
Acarbose^b^	–	–	–	–	667.40 ± 2.20

TCA, *Thymus canoviridis* aerial part, TCR, *Thymus canoviridis* root, TCEO, *Thymus canoviridis* essential oil.

^a^200 μg/mL.

^b^Standard compounds.

^c^IC_50_ values.

NA, Not Active.

Among the tested samples, the essential oil (TCEO) demonstrated the highest inhibition against both AChE (69.75 ± 1.14%) and BChE (69.61 ± 1.84%), showing comparable effectiveness to the standard drug galanthamine (AChE: 78.92 ± 1.04%; BChE: 78.22 ± 0.58%). The aerial extract (TCA) exhibited moderate BChE inhibition (33.93 ± 0.67%), while the root extract (TCR) showed weaker inhibition (13.89 ± 0.62%). None of the ethanol extracts showed measurable AChE activity at 200 μg/mL.

The α-glucosidase inhibition assay revealed that TCEO exhibited moderate activity with an IC_50_ value of 683.35 ± 3.75 μg/mL, comparable to the standard acarbose (IC_50_ = 667.40 ± 2.20 μg/mL). TCA displayed weaker inhibition (IC_50_ = 954.55 ± 1.48 μg/mL), and TCR showed no significant activity (IC_50_ > 1800 μg/mL).

None of the samples exhibited noteworthy activity against tyrosinase or urease at the tested concentration (200 μg/mL). In contrast, the standard inhibitors kojic acid and thiourea showed 95.05 ± 0.37% and 98.37 ± 0.40% inhibition against tyrosinase and urease, respectively.

The essential oil (TCEO) exhibited significantly higher BChE inhibition compared to both ethanol extracts (TCA and TCR) (*p* < 0.001). This difference was confirmed by one-way ANOVA and Tukey HSD *post-hoc* tests. The potent BChE inhibition by TCEO, despite its low phenolic/flavonoid content, suggests the presence of active volatile compounds selectively targeting BChE. Further phytochemical and *in silico* analyses are warranted to elucidate the underlying mechanism.

These results underline the potential of carvacrol-rich essential oil of *T. canoviridis* as a natural cholinesterase and α-glucosidase inhibitor, suggesting possible applications in managing neurodegenerative and metabolic disorders.

### 3.5 Antimicrobial activity of *T. canoviridis*

The ethanol extracts from the aerial (TCA) and root (TCR) parts, as well as the essential oil (TCEO) of *T. canoviridis*, were assessed for their antimicrobial potential against a panel of Gram-positive and Gram-negative bacteria and *Candida* species, using the microbroth dilution method (Table 5). Among the tested samples, the root extract (TCR) showed the strongest antibacterial activity, particularly against *Staphylococcus aureus* ATCC 29213 (MIC: 39.06 μg/mL) and *Candida tropicalis* ATCC 750 (MIC: 39.06 μg/mL). The aerial extract (TCA) also exhibited activity against *S. aureus* (MIC: 156.25 μg/mL) and *C. tropicalis* (MIC: 19.53 μg/mL), indicating notable anti-staphylococcal and antifungal properties.

**Table 5:** Minimum inhibitory concentrations (MIC, μg/mL) of ethanol extracts from aerial and root parts of *T. canoviridis* against tested microorganisms.

**Microorganisms**		**MIC Values (**μ**g/mL)**	
	**TCA**	**TCR**	**TCEO**
*P. aeruginosa* ATCC 27853	NA	NA	–
*E. coli* ATCC 25922	NA	NA	2500
*K. pneumoniae* ATCC 4352	NA	NA	2500
*P. mirabilis* ATCC 14153	NA	NA	2500
*S. aureus* ATCC 29213	156.25	39.06	5000
*S. epidermidis* ATCC 12228	312.50	1250	5000
*E. faecalis* ATCC 29212	NA	1250	NA
C. albicans ATCC 10231	NA	312.50	312.5
*C. parapsilosis* ATCC 22019	NA	NA	625
*C. tropicalis* ATCC 750	19.53	39.06	1250

TCA, *Thymus canoviridis* aerial part; TCR, *Thymus canoviridis* root; TCEO, *Thymus canoviridis* essential oil.

NA, No Activity. Standards; Cefuroxime-Na: 1.2 μg/ml for S. aureus ATCC 29213, Cefuroxime 9.8 μg/ml for S. epidermidis ATCC 12228, Amikacin 128 μg/ml for E. faecalis ATCC 29212, Ceftazidime 2.4 μg/ml for P. aeruginosa ATCC 27853, Cefuroxime-Na: 4.9 μg/ml for E. coli ATCC 25922 and K. pneumoniae 4352, Cefuroxime-Na 2.4 μg/ml for P. mirabilis ATCC 14153, Clotrimazole 4.9 μg/ml for C. albicans ATCC 10231, Amphotericin B 0.5 μg/ml for C. parapsilosis ATCC 22019, Amphotericin B 1 μg/ml for C. tropicalis ATCC 750.

The essential oil (TCEO), although rich in carvacrol, showed limited antimicrobial efficacy with relatively high MIC values (≥2500 μg/mL) for Gram-negative bacteria including *E. coli, K. pneumoniae*, and *P. mirabilis*. Against Gram-positive bacteria, TCEO was moderately active, with MICs of 5000 μg/mL against *S. aureus* and *S. epidermidis*, and 10,000 μg/mL against *E. faecalis*. Interestingly, TCEO exhibited the highest antifungal activity against *C. albicans* (MIC: 312.5 μg/mL), followed by *C. parapsilosis* (625 μg/mL) and *C. tropicalis* (1,250 μg/mL). This is consistent with the known antifungal properties of carvacrol-rich essential oils.

As positive controls, the following standard antimicrobials were used in parallel assays: Cefuroxime-Na (1.2 μg/mL for *S. aureus* ATCC 29213), Cefuroxime (9.8 μg/mL for *S. epidermidis* ATCC 12228), Amikacin (128 μg/mL for *E. faecalis* ATCC 29212), Ceftazidime (2.4 μg/mL for *P. aeruginosa* ATCC 27853), Cefuroxime-Na (4.9 μg/mL for *E. coli* ATCC 25922 and *K. pneumoniae* 4352), Cefuroxime-Na (2.4 μg/mL for *P. mirabilis* ATCC 14153), Clotrimazole (4.9 μg/mL for *C. albicans* ATCC 10231), Amphotericin B (0.5 μg/mL for *C. parapsilosis* ATCC 22019 and 1 μg/mL for *C. tropicalis* ATCC 750).

The observed stronger activity of the polar ethanol extracts, particularly TCR, against Gram-positive bacteria and *Candida* strains compared to the essential oil may be attributed to the high content of phenolic compounds such as rosmarinic acid, caffeic acid, and flavonoids, which possess synergistic antimicrobial mechanisms.

These findings suggest that both polar and non-polar fractions of *T. canoviridis* exhibit differential antimicrobial profiles, with ethanol extracts being more effective against Gram-positive pathogens and *Candida* spp., while the essential oil retains moderate antifungal activity. Such profiles support the potential use of *T. canoviridis* in developing food preservatives or natural antimicrobial agents for health-related applications.

## 4 Discussion

The present study aimed to comprehensively evaluate the chemical composition and biological activities of the essential oil and ethanol extracts obtained from the aerial and root parts of *T. canoviridis*, an endemic species to Türkiye. Essential oil profiling via GC-MS, phenolic compound quantification using LC-MS/MS, and *in vitro* biological activity assessments including antioxidant, enzyme inhibitory, and antimicrobial assays were conducted. The findings provide new insights into the phytochemical richness and bioactivity spectrum of this relatively understudied *Thymus* species. The exceptionally high carvacrol content observed in the essential oil, along with distinct phenolic profiles and notable bioactivities, underscore the potential of *T. canoviridis* as a valuable source of bioactive compounds for pharmaceutical, nutraceutical, and food industry applications. These results also contribute to the growing body of knowledge regarding the chemotaxonomic diversity within the *Thymus* genus and highlight the importance of regional phytochemical investigations.

In the essential oil composition of *T. canoviridis* analyzed in the present study, carvacrol was identified as the predominant compound, remarkably constituting 99.9% of the total content. Existing studies indicate that although a few species may exhibit carvacrol concentrations exceeding 90%, such instances are exceedingly rare. For example, Nooshkam et al. (25) reported that carvacrol was the major component of the essential oils of *Satureja khuzistanica* and *S. rechingeri* from Iran, ranging from 95.9% to 96.7%. Their investigation across multiple localities consistently revealed extraordinarily high carvacrol concentrations. Similarly, Figuérédo et al. (26) identified *Origanum dubium* (82.7%) and *O. minutiflorum* (86.1%) as the most carvacrol-rich oregano species among Mediterranean taxa, including those from Türkiye, Greece, Italy, and Morocco. Other species such as *O. compactum, O. dictamnus, O. onites*, and *O. vulgare* ssp. *hirtum* also contained high carvacrol levels, ranging from 55.9% to 76.4%. The first report on the essential oil composition of *T. canoviridis* was published in 1998, based on samples collected from Bayburt. In that study, carvacrol was identified as the major component, but at a relatively modest concentration of 29.51%, alongside significant amounts of geraniol (13.25%) and thymol (9.49%) (27). More recent investigations have demonstrated much higher carvacrol levels in this species: 52.87% in the study by Güven et al. (28) and 72.88% in that of Yigitkan and Firat (29). The striking variation between earlier and current results may be attributed to chemical polymorphism. For instance, Aboukhalid et al. (30) analyzed essential oils from 527 individuals of *O. compactum* collected from 88 different locations. Some samples contained no carvacrol at all, while others reached up to 96.3%. Pirbalouti et al. (31) also explored this phenomenon by examining ten populations of *S. khuzistanica* from various Iranian localities. Although all samples were carvacrol-dominant, concentrations varied significantly (42.5–94.8%), with higher levels generally observed in plants growing at elevated altitudes. In another study, Emrahi et al. (32) demonstrated that moderate water stress significantly increased carvacrol content (by up to 23%) in *O. vulgare* subspecies.

Türkiye is a leading exporter of oregano oil, derived from multiple species—including *Origanum, Thymus, Coridothymus, Thymbra, Satureja*, and *Lippia*—that are widely cultivated and processed for essential oil production. Carvacrol is considered the primary bioactive constituent in these species. Among them, *Origanum* taxa are often highlighted for their high carvacrol content, with concentrations reported to reach up to 84% in Turkish species. However, certain *Thymus* species such as *T. migricus* and *T. kotschyanus* var. *glabrescens* have also been found to be rich in carvacrol, with levels up to 78% (33). To the best of our knowledge, the present study is the first to report a *Thymus* species with carvacrol content as high as 99.9%, possibly making *T. canoviridis* the first known *Thymus* taxon composed almost entirely of a single compound. Such monocomponent essential oils are exceptionally uncommon. As a notable exception, Tsuruoka et al. (34) identified 4aα,7α,7aα-nepetalactone as the sole compound in the essential oil of *Nepeta sibirica*. Extensive studies have characterized the essential oil compositions of various *Thymus* species from Türkiye, revealing considerable chemotypic diversity. For instance, Boga et al. (11) reported that camphor was predominant in *T. convolutus* (12.7%) and *T. sipyleus* (13.1%), while *T. fallax* was rich in bicyclogermacrene (21.5%). In *T. kotschyanus* var. *kotschyanus*, carvacrol (48.5%) and thymol (22.5%) were the main constituents. In contrast, *T. haussknechtii* was found to contain 28.2% carvacrol. Meanwhile, Küçükbay et al. (35) reported that *T. kotschyanus* var. *kotschyanus* primarily consisted of geraniol (55.0–59.1%) and geranyl acetate (27.1–28.8%). Other varieties, such as *T. kotschyanus* var. *eriophorus* and *T. kotschyanus* var. *glabrascens*, were characterized by carvacrol (57.2%) and its biosynthetic precursor p-cymene (11.0%). Additionally, *T. serpyllum* was shown to contain thymol (1702 mg/100 g) and carvacrol (179 mg/100 g) as major components (36). In another study, the endemic *T. argaeus* was reported to be rich in linalool, α-terpineol, and linalyl acetate (37). These findings collectively underscore the remarkable variability in essential oil profiles among *Thymus* species, which is influenced by a multitude of genetic, geographic, and environmental factors.

In the present study, LC-MS/MS analysis revealed that rosmarinic acid was the major phenolic constituent in both aerial and root ethanol extracts of *T. canoviridis*, accompanied by notable amounts of apigenin, quinic acid, and other hydroxycinnamic acid derivatives. A previous UPLC-MS/MS study investigated the water and methanol extracts of *T. canoviridis* leafy flowers, revealing that the methanol extract was notably richer in phenolic compounds. In comparison with the current study, there are considerable consistencies in the phytochemical profiles. Specifically, secondary metabolites such as apigenin, rosmarinic acid, and quinic acid were consistently identified across all tested extracts. Furthermore, compounds like naringenin, caffeic acid, fumaric acid, and ferulic acid were present in three out of the four extracts analyzed (28). Among the *Thymus* species naturally growing in Türkiye, rosmarinic acid stands out as a frequently occurring and dominant phenolic constituent. For instance, *T. nummularius* was reported to contain a particularly high amount of rosmarinic acid, reaching 131.899 ± 6.463 mg/g dry extract (15). Similarly, *T. pectinatus* and *T. convolutus* were also identified as rosmarinic acid-rich species, with this compound being the major phenolic in both (38). In another study, *T. cariensis* (2501.3 ± 178.34 μg/g), *T. praecox* subsp. *grossheimii* (2166.60 ± 154.48 μg/g), and *T. pubescens* (2499.32 ± 178.20 μg/g) were also shown to accumulate considerable levels of rosmarinic acid in their aerial parts (13). Additionally, other species such as *T. leucostomus* (34.8%), *T. brachychilus* (15.801 μg/g), *T. argaeus* (6.574 μg/g), *T. fallax* (9631.71 ± 686.74 μg/g), *T. haussknechtii* (10579.5 ± 754.32 μg/g), *T. kotschyanus var. kotschyanus* (1800.18 ± 128.35 μg/g), and *T. sipyleus* (2924.30 ± 208.50 μg/g) were also characterized as rosmarinic acid-rich taxa (11, 37–40).

In our study, the total phenolic content of *T. canoviridis* extracts was notably higher than the total flavonoid content, with the aerial part extract exhibiting slightly greater phenolic accumulation compared to the root extract. In the study by Köksal et al. (41), the ethanol extract of *T. vulgaris* was found to contain 158 μg gallic acid/mg extract of total phenolics and 36.6 μg quercetin/mg extract of total flavonoids. Similarly, Ertaş et al. (15) reported that in *T. nummularius*, the total phenolic content was higher than the total flavonoid content, consistent with the results of the present study. Niculae et al. (42) also demonstrated high total phenolic content in a 70% ethanol extract of *T. marschallianus*, further supporting the trend seen across different *Thymus* species.

### 4.1 Antioxidant activity

In the present study, both ethanol extracts and the essential oil of *T. canoviridis* exhibited substantial antioxidant activity, as evidenced by their performance in DPPH, ABTS, and CUPRAC assays, with the aerial and root extracts showing differential strengths across methods, and the essential oil demonstrating remarkable ABTS scavenging capacity despite its limited DPPH activity. In terms of antioxidant activity, the root extract (DPPH IC_50_: 24.32 ± 0.84 μg/mL) exhibited stronger scavenging potential than the aerial part extract (DPPH IC_50_: 33.08 ± 0.54 μg/mL). For the ABTS assay, the aerial part extract (ABTS IC_50_: 8.36 ± 0.05 μg/mL) outperformed the root extract (ABTS IC_50_: 9.72 ± 0.08 μg/mL). The root extract also demonstrated superior reducing power in the CUPRAC assay (A_0.5_: 9.99 ± 0.02 μg/mL) compared to the aerial parts (A_0.5_: 10.59 ± 0.04 μg/mL). However, both extracts showed weaker activity than synthetic standards such as BHA, α-tocopherol (α-TOC), and BHT. Overall, the root extract showed better performance in both DPPH scavenging and cupric ion reduction, while the aerial extract showed better ABTS scavenging activity. Despite being less potent than synthetic standards, both extracts exhibited considerable antioxidant potential, particularly in the ABTS assay. The essential oil of *T. canoviridis* (TCEO) had a significantly higher DPPH IC_50_ (273.42 ± 1.65 μg/mL), indicating relatively weak activity compared to BHA (7.88 ± 0.20 μg/mL), α-TOC (16.30 ± 0.79 μg/mL), and BHT (58.86 ± 0.50 μg/mL). In contrast, TCEO exhibited a remarkably strong ABTS IC_50_ value (0.43 ± 0.02 μg/mL), outperforming all tested synthetic standards. CUPRAC activity was also high (A_0.5_: 3.36 ± 0.07 μg/mL), though not superior to BHA and BHT. These results emphasize that while TCEO has limited efficacy in DPPH scavenging, it is highly effective in ABTS scavenging and cupric ion reduction. The discrepancies across assays highlight the importance of using multiple antioxidant evaluation methods to assess different modes of action. In line with the current findings, Üstüner et al. (43) reported that the ethanol extract of *T. sipyleus* subsp. *rosulans* inhibited 50% of DPPH radicals at 104.91 μg/mL. Akin and Saki (44) showed that ethanol extract from *T. vulgaris* had 77% inhibition at 400 μg/mL, while Köksal et al. (41) noted a DPPH IC_50_ of 12.1 μg/mL. The methanol extract of *T. nummularius* exhibited exceptional DPPH activity (IC_50_: 5.73 μg/mL) exceeding that of BHT and α-TOC (15). These results align well with the current study, where both *T. canoviridis* extracts displayed notable DPPH scavenging potential. Eroglu Özkan et al. (13) demonstrated DPPH IC_50_ values of 34.97, 51.44, and 41.80 μg/mL for *T. cariensis* and the aerial/root extracts of *T. pubescens*, respectively, aligning with the present values for *T. canoviridis*. Regarding ABTS activity, the IC_50_ values of 8.36 and 9.72 μg/mL for aerial and root extracts were better than that of α-TOC. Küçükaydin et al. (12) reported comparable results for *T. cariensis* and *T. cilicicus*. Köksal et al. (41) reported a higher ABTS IC_50_ of 54.08 μg/mL for *T. vulgaris*, whereas Bendjabeur et al. (45) found 7.44 μg/mL for *T. algeriensis*. Likewise, Boga et al. (11) showed values between 6.48 and 18.75 μg/mL for five *Thymus* species, with the most active being the root extract of *T. haussknechtii*. In the CUPRAC assay, both extracts of *T. canoviridis* were more effective than α-TOC. The ethanol extract of *T. algeriensis* showed an A_0.5_ of 19.40 μg/mL (45), while *T. nummularius* also surpassed α-TOC in efficacy (15). Boga et al. (11) and Eroglu Özkan et al. (13) further supported this trend, reporting high CUPRAC activities in various *Thymus* taxa. In the present study, the essential oil of *T. canoviridis* demonstrated notable inhibitory activity against the α-glucosidase enzyme, with an IC_50_ value of 683.35 ± 3.75 μg/mL—nearly comparable to that of the reference compound acarbose (IC_50_ = 667.40 ± 2.20 μg/mL). This finding highlights the potential of *T. canoviridis* essential oil, which comprises 99.9% carvacrol, as a promising candidate for further antidiabetic investigations.

### 4.2 Enzyme inhibition activity

In the present study, the essential oil of *T. canoviridis*, characterized by an exceptionally high carvacrol content (99.9%), exhibited a strong α-glucosidase inhibitory effect, with an IC_50_ value comparable to that of the reference drug acarbose. For instance, Salazar et al. (46) reported that *Origanum vulgare* essential oil, containing high levels of thymol and carvacrol, effectively inhibited α-glucosidase, and synthetic derivatives of these compounds exhibited potent activity through a mixed-type inhibition mechanism. Similarly, Singh et al. (47) emphasized the relevance of oxygenated monoterpenes like thymol and carvacrol in α-glucosidase inhibition. Ali (48) investigated the essential oil of *T. vulgaris*, composed primarily of thymol (55.88%), linalool (13.71%), carvacrol (8.36%), and p-cymene (6.00%). The oil showed potent α-glucosidase inhibitory activity, with an IC_50_ value of 125.1 ± 4.25 μg/mL, further supporting the bioactivity of carvacrol-rich compositions.

In our study, *T. canoviridis* essential oil displayed selective cholinesterase inhibition, showing no significant activity against acetylcholinesterase (AChE) but exhibiting a strong inhibitory effect on butyrylcholinesterase (BChE), with 69.61 ± 1.84% inhibition at 200 μg/mL. The notable BChE inhibition shown by TCEO can be ascribed to its rich content of oxygenated monoterpenes such as carvacrol and borneol, which may interact favorably with the enzyme's broader active site, consistent with earlier findings on *Thymus* species. Regarding cholinesterase inhibition, our findings align with earlier literature reporting selective inhibition patterns. While *T. canoviridis* essential oil exhibited no notable activity against acetylcholinesterase (AChE), it significantly inhibited butyrylcholinesterase (BChE) with 69.61 ± 1.84% inhibition at 200 μg/mL. This pattern has also been observed in other *Thymus* taxa. For example, Boga et al. (11) reported that the ethanol extract from the aerial parts of *T. fallax* displayed notable BChE inhibitory activity (34.48 ± 0.60%) but was inactive against AChE and tyrosinase. Kindl et al. (49) investigated the AChE inhibition profile of various *Thymus* species, including *T. longicaulis, T. praecox* subsp. *polytrichus, T. pulegioides, T. serpyllum* subsp. *serpyllum, T. striatus*, and *T. vulgaris*, using the Ellman colorimetric method. The extracts exhibited dose-dependent AChE inhibition, ranging from 10 to 28%, 23 to 39%, and 64 to 86% at concentrations of 0.25, 0.5, and 1 mg/mL, respectively. Similarly, Ekin et al. (50) found that the ethanol extract of *T. zygioides* var. *lycaonicus* showed no activity against AChE at 2000 μg/mL but displayed significant BChE inhibition (30.92 ± 1.44%). Orhan et al. (51) also reported that the ethanol extract of *T. praecox* subsp. *caucasicus* var. *caucasicus* was inactive against AChE, even at 1000 μg/mL, corroborating the selectivity trend seen in our results. Collectively, the findings support the growing body of evidence that *Thymus* species, particularly their essential oils or ethanol extracts, may preferentially target BChE or α-glucosidase over AChE, suggesting selective enzyme modulation that could be valuable in managing neurodegenerative or metabolic disorders.

### 4.3 Antimicrobial activity

In the present study, the antimicrobial potential of *T. canoviridis* was assessed through the broth microdilution method, following the guidelines of the Clinical and Laboratory Standards Institute (CLSI). The Minimum Inhibitory Concentration (MIC) values were determined against a panel of ten pathogenic strains, including six bacterial (Gram-positive and Gram-negative) and four fungal species. These included *Staphylococcus aureus, Staphylococcus epidermidis, Enterococcus faecalis, Escherichia coli, Pseudomonas aeruginosa, Klebsiella pneumoniae, Proteus mirabilis, Candida albicans, Candida parapsilosis*, and *Candida tropicalis*. Among the tested extracts, the ethanol extract of the root part (TCR) showed the most pronounced antibacterial activity against *S. aureus*, with a MIC value of 39.06 μg/mL, while the aerial part (TCA) demonstrated moderate inhibition at 156.25 μg/mL. Notably, both aerial and root ethanol extracts exhibited strong antifungal activity against *C. tropicalis*, with MIC values of 19.53 μg/mL and 39.06 μg/mL, respectively, indicating remarkable sensitivity of this fungal strain to *T. canoviridis* phytochemicals. These findings are consistent with previous literature that underscores the antimicrobial potential of various *Thymus* species. For example, in a study by Oztürk et al. (52), the methanol extract of *T. fallax* demonstrated broad-spectrum antibacterial activity with MICs ranging from 31.25 to 500 μg/mL against strains such as *Arthrobacter atrocyaneus, Bacillus sphaericus, Enterobacter hormaechei, Staphylococcus cohni cohni, Pseudomonas syringae*, and *Kocuria rosea*. Afonso et al. (53) reported strong activity of *T. herba-barona, T. pseudolanuginosus*, and *T. caespititius* against *S. aureus*, with remarkably low MIC values of 0.6, 1.6, and 3.5 μg/mL, respectively, suggesting that certain *Thymus* species possess compounds with potent Gram-positive antibacterial activity. Similarly, Naz et al. (54) demonstrated that *T. linearis* extracts showed inhibitory zones ranging from 12 to 23 mm against *Salmonella typhi, S. aureus*, and *Citrobacter freundii*. Boga et al. (11) emphasized the antifungal efficacy of *T. convolutus* and *T. haussknechtii*, particularly against *C. tropicalis*, with MIC values similar to those observed in the current study (19.53 μg/mL). Furthermore, Eroglu Özkan et al. (13) reported comparable antifungal activity of *T. cariensis, T. praecox* subsp. *grossheimii*, and *T. pubescens* against *C. tropicalis*, with MICs ranging from 19.53 to 78.12 μg/mL. Collectively, these results support the relevance of *T. canoviridis* as a promising natural antimicrobial agent, particularly in the context of fungal infections and Gram-positive bacterial pathogens such as *S. aureus*.

Given the essential oil of *T. canoviridis* contains carvacrol at a remarkably high level (99.9%), it can be considered as a promising natural bioactive agent. Due to carvacrol's well-documented antimicrobial, antifungal, antioxidant, and anti-inflammatory properties, the essential oil may find practical applications in food preservation, dermatological preparations, natural pesticides, and veterinary products as a natural and eco-friendly alternative to synthetic agents.

## 5 Conclusion

The present study provides a detailed phytochemical and biological evaluation of *T. canoviridis*, emphasizing its potential as a valuable ingredient for food and nutraceutical applications. The essential oil was found to be remarkably rich in carvacrol (99.9%), a compound widely recognized for its antimicrobial, antioxidant, and preservative properties. Ethanol extracts of both aerial and root parts demonstrated notable phenolic richness, particularly in rosmarinic acid and apigenin, contributing to their strong antioxidant capacities. The extracts and essential oil exhibited selective enzyme inhibitory activities, especially against butyrylcholinesterase and α-glucosidase, which may offer added value in the context of functional food formulations aimed at metabolic and cognitive health support. Moreover, the pronounced antifungal activity against *Candida tropicalis* and antibacterial effect against *Staphylococcus aureus* underline the potential of *T. canoviridis*-derived preparations as natural preservatives in food systems.

Overall, this work represents the first integrated investigation of both volatile and non-volatile fractions of *T. canoviridis*, highlighting its distinctive phytochemical and bioactivity profile. These findings contribute significantly to the limited scientific knowledge on endemic Anatolian *Thymus* species and establish a foundation for the future valorization of *T. canoviridis* as a promising functional ingredient in food and health-promoting applications.

## Data Availability

The original contributions presented in the study are included in the article/Supplementary material, further inquiries can be directed to the corresponding author/s.
