# Supplementary material for: Exploring the metabolite composition and biological activities of Thymus canoviridis via volatile and non-volatile fraction analysis
Source: Front Nutr. 2026 Jan 14;12:1675586. doi: 10.3389/fnut.2025.1675586 (PMC12851589; doi:10.3389/fnut.2025.1675586)
Supplement: Supplementary file 1 [file Supplementary_file_1.docx]

**Fig 1.** TIC Chromatogram of *Thymus canoviridis* essential oil
